# *Notes From the Field*: Enhanced Identification of Tobacco Use Among Adult Medicaid Members — King County, Washington, 2016–2023

**DOI:** 10.15585/mmwr.mm7407a2

**Published:** 2025-03-06

**Authors:** Amber K. Sabbatini, Austin Craig, Eli Kern, Susan Hernandez, Caroline Brazeel, Alexandra Kearly, Madison Hluchan, Orobosa Idehen, Elizabeth Courtney-Long, Corinne Husten, Brian S. Armour

**Affiliations:** ^1^University of Washington, Seattle, Washington; ^2^Public Health – Seattle & King County, Seattle, Washington; ^3^Association of State and Territorial Health Officials, Arlington, Virginia; ^4^Office on Smoking and Health, National Center for Chronic Disease Prevention and Health Promotion, CDC.

**Summary**

**What is already known about this topic?**

Among adults covered by health insurance, prevalence of tobacco use is highest among Medicaid members. Reimbursement claims data alone can underestimate tobacco use.

**What is added by this report?**

During 2016–2023, supplementing reimbursement claims data with tobacco use information from Medicaid enrollment identified 14,163 (14.0%) additional Medicaid members, including a higher proportion of men and those who did not have a diagnosed chronic condition.

**What are the implications for public health practice?**

Identifying tobacco use during Medicaid enrollment enhances public health data collection and provides opportunities to better diagnose and treat tobacco use.

In 2021, tobacco use prevalence among Medicaid members nationwide was 28.1%, compared with 16.2% among those with private insurance (*1*). Medicaid members experience a higher prevalence of tobacco-related diseases as well as gaps in receiving effective tobacco cessation therapies (*2*,*3*), which results in Medicaid spending of approximately $68 billion annually on smoking-related diseases (*4*). Identifying Medicaid members who use tobacco and providing cessation interventions can improve health and reduce health care costs.

## Investigation and Outcomes

Washington is one of five states* that collect tobacco use data on Medicaid eligibility forms. To examine how linking enrollment data with tobacco use and smoking cessation claims data might improve identification of Medicaid members who use tobacco, self-reported tobacco use data collected through the Washington Health Benefit Exchange (HBE),^†^ an online marketplace for health insurance, were combined with reimbursement claims data to identify a cohort of King County, Washington Medicaid members aged 18–64 years who use tobacco. Those enrolled through HBE^§^ or with claims related to tobacco use during 2016–2023 were included in this analysis. To ensure adequate time for diagnosis of comorbidities, Medicaid members were required to be enrolled ≥ 7 months in any given year.

The primary outcome was the ascertainment of usage data on tobacco products among Medicaid members. Adults responding affirmatively on the enrollment form to using tobacco products (excluding e-cigarettes and vape products) more than four times weekly during the preceding 6 months were classified as current tobacco users in the HBE data. In claims data, enrollees were considered to use tobacco if they had a diagnosis of tobacco use, an *International Classification of Diseases, Tenth Revision, Clinical Modification* (ICD-10-CM) or procedure code for tobacco cessation services, or a pharmacy claim for nicotine replacement therapy or varenicline.^¶^ Descriptive analyses compared characteristics of persons identified in claims versus enrollment data. The state of Washington Institutional Review Board review determined that the project meets the criteria delineated in 45 CFR 46.102(l)(2) as surveillance. CDC did not perform an independent institutional review board determination.

Among a Medicaid member population of 511,154, a total of 101,060 (19.8%) were identified as using tobacco based on self-reported HBE data, claims criteria, or both (Table). Neither data source alone identified all Medicaid members who used tobacco: 14,163 (14.0%) were identified only in HBE data, 51,534 (51.0%) were identified only through claims data, and 35,363 (35.0%) were identified in both HBE and claims data.

**TABLE:** Characteristics of Medicaid members who use tobacco, identified through self-reported data collected during enrollment in the Health Benefits Exchange* and in reimbursement claims — King County, Washington, 2016–2023

Characteristic	Source of Medicaid member tobacco use data, no. (%)^†^		
	HBE, claims,^§^ or both^ ^N = 101,060^¶^	HBE only^§ ^n = 14,163	Claims only^§ ^n = 51,534
**Age group, yrs**			
18–24	**8,965 (8.9)**	1,506 (10.6)	6,104 (11.8)
25–34	**28,444 (28.1)**	5,026 (35.5)	15,078 (29.3)
35–44	**26,533 (26.3)**	3,494 (24.7)	13,483 (26.2)
45–54	**18,292 (18.1)**	2,168 (15.3)	8,682 (16.8)
55–64	**18,826 (18.6)**	1,969 (13.9)	8,187 (15.9)
**Sex**			
Female	**41,889 (41.4)**	4,564 (32.2)	23,485 (45.6)
Male	**59,671 (59.0)**	9,647 (68.1)	28,355 (55.0)
Race and ethnicity**			
American Indian or Alaska Native	**6,877 (6.8)**	817 (5.8)	3,425 (6.6)
Asian	**7,705 (7.6)**	1,254 (8.9)	3,993 (7.7)
Black or African American	**20,945 (20.7)**	2,249 (15.9)	11,940 (23.2)
Native Hawaiian or Pacific Islander	**6,253 (6.2)**	935 (6.6)	3,501 (6.8)
White	**60,958 (60.3)**	8,285 (58.5)	29,599 (57.4)
Hispanic or Latino	**11,964 (11.8)**	1,425 (10.1)	7,164 (13.9)
Unknown	**4,271 (4.2)**	920 (6.5)	2,240 (4.3)
**Language other than English**	**5,024 (5.0)**	659 (4.7)	3,061 (5.9)
**Chronic condition** ^††^			
No chronic conditions	**16,232 (16.1)**	7,365 (52.0)	5,577 (10.8)
Physical health condition	**51,885 (51.3)**	2,248 (15.9)	28,726 (55.7)
Behavioral health condition	**77,588 (76.8)**	6,075 (42.9)	42,025 (81.5)

**Abbreviation**: HBE = Health Benefits Exchange.

* The Washington HBE is a public-private partnership responsible for the operation of Washington Healthplanfinder, an online marketplace to find qualified health plans. https://www.wahbexchange.org

^†^ Numbers in each category might not sum to the total because of missing data.

^§^ Estimates in each category might not sum to 100% because of rounding.

^¶^ A total of 35,363 members were identifiable by both HBE and claims data.

** Data for some groups are not mutually exclusive; thus, total sums might exceed 100%.

^††^ Physical health conditions were defined using Centers for Medicare & Medicaid Services Chronic Conditions Data Warehouse algorithms, excluding depression. Behavioral health conditions were defined using measure specifications developed by the Washington State Department of Social and Health Services for Medicaid claims data.

Characteristics of Medicaid members who used tobacco differed by data source. Compared with the claims-only data, a larger proportion of persons in HBE-only data were aged 25–34 years (35.5% versus 29.3%), male (68.1% versus 55.0%), and had no chronic conditions (52.0% versus 10.8%). Claims-only data identified a larger proportion of Medicaid members who were female (45.6% versus 32.2%), Black or African American (23.2% versus 15.9%), or Hispanic or Latino (13.9% versus 10.1%), and who had physical (55.7% versus 15.9%) or behavioral health conditions (81.5% versus 42.9%).**

## Preliminary Conclusions and Actions

These findings suggest that asking persons about their tobacco use at the time of health insurance enrollment represents an opportunity to proactively intervene with cessation services, most notably for men and persons not yet experiencing a chronic condition to potentially lower their risk for developing these conditions. Using multiple data streams to identify Medicaid members who use tobacco enhances public health surveillance (*5*) and has the potential to improve identification of tobacco users and strengthen and expand cessation treatment.

## Footnotes

* Arkansas, Indiana, Missouri, Utah, and Washington.

† The Washington HBE is a public-private partnership responsible for the operation of Washington Healthplanfinder, an online marketplace to find qualified health plans. https://www.wahbexchange.org

§ Adults eligible for both Medicaid and Medicare, those receiving services via the aged, blind, and disabled pathway, and those receiving long-term care services do not enroll through HBE but through an alternative platform called Washington Connection run by the Washington Department of Social and Human Services. Self-reported tobacco use is not available for these beneficiaries.

¶ ICD-10-CM codes for tobacco use (Z72.0, F17.2xx, O99.33x, T65.21[124], T65.22[124], or T65.29[124]); ICD-10-CM code for tobacco use counselling Z71.6; Current Procedural Terminology (CPT)/Healthcare Common Procedure Coding System (HCPCS) procedure code for tobacco use counseling (99406, 99407, S9453, 4000F, or 4001F); National Drug Codes (NDCs) for nicotine replacement therapy; NDC for varenicline; HCPCS procedure code tobacco screening: G9902, G9906, or G9908. This definition includes adults who currently use tobacco and specifically excludes diagnosis codes for former tobacco use, a history of tobacco use, and exposure to secondhand smoke.

** Physical health conditions were defined by using specifications provided by the Chronic Conditions Data Warehouse (https://www2.ccwdata.org/web/guest/condition-categories-chronic); behavioral health conditions were defined by using adapted specifications provided by the Washington State Department of Health and Social Services for Mental Health Service Rate and Substance Use Disorder Treatment Rate measures. https://www.dshs.wa.gov/ffa/research-and-data-analysis/measure-specifications
